# Investigation of mechanical properties and micromechanisms of saline soil modified with synthetic fibers

**DOI:** 10.1371/journal.pone.0329941

**Published:** 2025-08-14

**Authors:** Zhixin Liu, Dongmei Chen, Jili Qu

**Affiliations:** 1 University of Shanghai for Science and Technology, School of Environment and Architecture, Shanghai, China; 2 Kashi University, School of Civil Engineering, Xinjiang Kashi, China; University of Sharjah, UNITED ARAB EMIRATES

## Abstract

To study the mechanical properties and microscopic morphology of salt-affected soil after being improved by fiber types and contents, the article analyzes the unconfined compressive strength and shear strength of the sulfate-affected soil in Kashi, Xinjiang, China, which four different fiber contents have improved. Some samples are tested by scanning electron microscope (SEM) and nuclear magnetic resonance (NMR) microanalysis. The article selects the sample with the highest improved unconfined compressive strength for dry-wet cycling and dissolution test. The results show that polypropylene, polyester, and glass fiber can increase the maximum dry density of salt-affected soil. The unconfined compressive strength of the soil with 1% polyester fiber and 8% silica fume reinforcement is the highest, which is 1.98 times that of the original soil. The unconfined compressive strength of the soil with 1% polyester fiber reinforcement is the largest, 1.43 times that of the original soil. The unconfined compressive strength of the soil with 5% and 7% glass fiber reinforcement is relatively large, 1.56 and 1.57 times that of the original soil, respectively. The cohesion of the original soil is the largest. The internal friction angle of the soil with 6% glass fiber reinforcement is the largest. In addition, the addition of synthetic fibers can significantly reduce the dissolution coefficient of salt-affected soil, especially glass fibers. Through SEM and NMR analysis, it is found that fibers form a good clamping action with soil particles, and some fibers have a tight bond with the soil, reducing the porosity of the soil.

## Introduction

Saline soils(ST) are widely distributed around the global, especially in arid and semi-arid regions, where salt accumulates in the soil as a result of long-term exposure to the effects of climate change [1–3]. The formation of saline soils is closely related to the geographical, climatic and hydrological conditions of the region, and the accumulation of soil salts is particularly significant in river valleys, near lakes and in areas with a high water table. In these areas, evaporation is greater than precipitation, causing dissolved salts in the groundwater to rise to the surface and gradually be deposited, ultimately forming saline soils [4–6]. From an engineering point of view, the presence of excess salts can severely deteriorate the physical and mechanical properties of soils, thus posing significant challenges for infrastructure development and geotechnical applications [7–9].

High salinity promotes dispersion of clay particles, reduces inter-particle bonding, and leads to a significant reduction in shear strength, compressive and load-bearing capacity, thus affecting soil structure [10]. In addition, saline soils are prone to volumetric instability under wetting-drying and freeze-thaw cycles, leading to differential settlement and structural damage [11]. These effects are further exacerbated by the fact that salt crystallization and hydration can disrupt soil structure and accelerate strength loss. Understanding and improving the engineering behavior of saline soils is therefore critical to ensuring the stability and durability of civil infrastructure in affected areas [12].

As a typical inland arid area, Kashi region of Xinjiang is affected by special geographic location and climatic conditions, saline soils are widely distributed, with high salt content, types dominated by chloride salts and sulfate salts, with obvious high alkalinity and strong expansion and contraction [13]. As evaporation in the region is much larger than precipitation, the water table is shallow, and dissolved salts are easy to rise with capillary water and accumulate on the surface, making the soil structure loose, decreasing strength, deteriorating deformation characteristics, and showing obvious uneven settlement, cracking and insufficient bearing capacity and other engineering problems [14]. Kashi saline soil not only has high salt content, and its engineering properties are easily affected by environmental changes, especially in the rainy season or after irrigation is prone to wet subsidence, disintegration and other destructive deformation [15]. A large number of engineering practices have shown that the low shear strength, high compressibility and small elastic modulus of this type of soil in its natural state make it difficult to meet the load-bearing requirements for the construction of roads, sites, pipelines and building foundations. Therefore, how to effectively improve the engineering performance of saline soils in Kashi and enhance their stability and durability has become an important technical problem to be solved in regional engineering construction [16].

In the past decades, many soil stabilization techniques have been explored to improve the geotechnical properties of saline soils, and two main approaches have been investigated: physical amendment techniques and chemical amendment techniques [17–22]. Physical improvement techniques are primarily achieved through replacement and compaction [23]. Soil replacement wastes a large amount of land resources and does not meet the requirements of ecological protection. In addition, silt soil has low plasticity, poor cohesion and low strength, which makes it difficult to reach the required compaction level. Therefore, chemical improvement technology has been widely used in the field of geotechnical engineering [24], Cement, lime, and asphalt are the most commonly used chemical improvement materials [25]. Several scholars have previously studied changes in the mechanical strength of saline soils using chemical methods, Zhu [26] obtained the conclusion that the UCS of the improved soil increased with the increase of ZM concentration and maintenance time by adding six new aqueous polymers with different contents of ZM. Huang [27]studied the unconfined compressive strength, soaking stability and permeability of saline soils with different cement additions (0%, 3%, 5%, 7%, 10%) and compaction factors (92%, 95%, 98%) and found that the increase of cement additions and compaction factors significantly increased the compressive strength and soaking stability of saline soils, reduced the rate of mass loss, and improved the residual strength ratio, especially when the cement additions reached 7% and above, and the compaction factor was 98%, saline soils had good soaking stability. In particular, when the cement addition amount reaches 7% and above, and the compaction coefficient is 98%, the saline soil has good soaking stability.Gu [28] used polyacrylamide (PAM) as a water-soluble polymer to improve the compaction properties of highway roadbed fill based on saline soils and the cracking resistance of saline soil crusts, and investigated the compressibility, microstructure, and cracking patterns of untreated and PAM-treated saline soils. The results showed that the liquid and plastic limits at the surface and depth of the saline soil specimens increased significantly with the increase of PAM mass fraction, the maximum dry density decreased with the increase of PAM mass fraction and plasticity, and there was no significant change in the optimum water content of the 2 saline soil types. Quantitative analysis of the cracking patterns revealed that the increase in PAM reduced the shrinkage strain and defects or pores in the saline soils.However, chemical methods often involve high costs, carbon emissions or secondary environmental impacts. As environmental problems such as global warming intensify, there is growing interest in developing sustainable and environmentally friendly soil amendment technologies [29].

Through the analysis of some existing improved materials [30–37],synthetic fibers have the advantages of low sensitivity, high strength, and high yield compared with natural fibers [38], the use of synthetic fibers as reinforcement materials is gaining attention in emerging strategies. Synthetic fibers such as polypropylene (PPF), polyester (PETF), polyethylene (PEF), and glass fibers (GF) have the advantages of high tensile strength, chemical resistance, and ease of integration into the soil matrix [39,40]. These fibers were effective in enhancing cohesion, ductility and crack resistance of the treated soils. It was shown that cohesion increased in polypropylene fiber lime soil and jute lime soil, and decreased in the order of straw fiber lime soil and wheatgrass lime soil. Chen Lei et al. studied the addition of different dosages of polypropylene fibers to lime-improved expansive soils, and found that the addition of the appropriate amount of fibers can inhibit soil expansion and improve the compressive strength and shear strength of the soil [41]. SOHEIL [42]used geogrid to improve the soil ductility, the study examined the effect of geogrid and lime treatment of soil UCS, destructive strain, cut line modulus, deformation index, rebound modulus, bulk modulus and shear modulus, the test results show that the addition of geogrid and lime significantly improved the geotechnical properties of cohesive soils.In addition, several studies have shown that fiber reinforcement can mitigate the adverse effects of salt on soil strength and improve long-term durability under environmental stresses [43].Despite these advances, the application of synthetic fibers in stabilizing high-salinity soils under multi-environmental stress conditions (e.g., dry–wet cycling, salt dissolution) remains insufficiently studied, particularly in regions such as Kashi.

To address this research gap, this study aims to systematically investigate the mechanical and microstructural improvement effects of industrially recycled or waste-derived synthetic fibers (PPF, PETF, PEF, and GF) on the saline soils in the Kashi region. A series of laboratory tests, including unconfined compressive strength, direct shear strength, dry–wet cycling, and salt dissolution–trapping tests, were conducted to assess performance changes. In addition, microstructural analyses using scanning electron microscopy (SEM) and nuclear magnetic resonance (NMR) were carried out to reveal the internal mechanisms of fiber–soil interaction.This research provides a novel, low-carbon, and eco-friendly stabilization strategy for saline soils in arid and semi-arid regions, and offers a technical reference for large-scale engineering applications in northwestern China and similar contexts worldwide.

## Test Materials and methods

### Test materials

**Fig 1:**
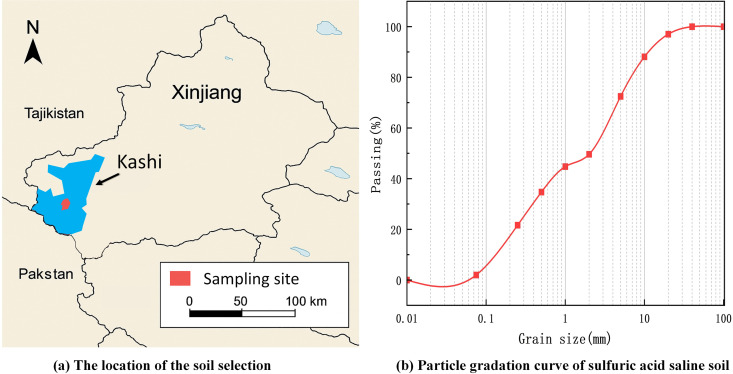
Soil selection location and grading curve.

**Fig 2:**
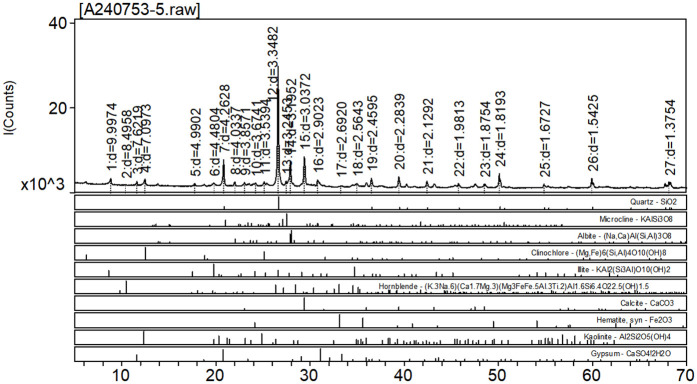
Chemical composition of saline soil.

The soil was selected from the Kashi region of Xinjiang, China, and the specific location was shown in Fig 1(a), and the selected saline soil was sulfate saline soil, whose basic physical indexes and gradation curves are shown in Table 1 and Fig 1(b). The chemical composition of saline soil is detected by Shanghai Chongyan Technology. The specific composition is shown in Table 2 and Fig 2, The main chemicals contained in soil are CaCO_3_, CaSO_4_, and so on.

**Table 1:** The primary physical indexes of sulfuric acid saline soil.

plastic limit (%)	liquid limit (%)	natural moisture content (%)	optimum moisture content (%)	maximum dry density (g/cm^3^)	specific gravity
16	35	4.22	14	1.96	2.65

**Table 2:** Chemical composition of saline soil (relative mineral mass content ×10^−2^).

Qtz	Kfs	Ab	C2S	Ank	Dol	Cal	Hem	Chl	It	Tm	Gyp
43.5	2.3	12.7	0.0	0.0	1.2	16.8	1.3	7.0	13.1	0.1	1.9

**Fig 3:**
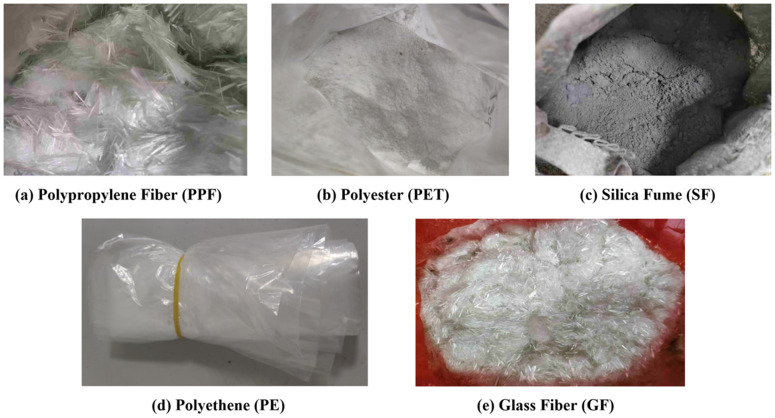
Experimental materials.

Synthetic fibers used are polypropylene (PP), polyester (PET), polyethene (PE), glass fiber (GF), and PP, which are purchased from Sanxin Company. PET is purchased from Kexinda Polymer Material Company, and the product specification is 300 micrometres, 50 mesh, the density is 1.38G/CM^3^, PE is used in the industry to produce plastic bags that are usable for life., GF specification is 3 mm, and the product brand is 5301-A. Other materials, silica fume (SF) (cement dosage 6% ~ 12%), were purchased from Yixiang New Material, The chemical composition and surface morphology of the employed synthetic fibers have been widely reported in existing literature. PP, PE and PET are thermoplastic polymers composed of long-chain hydrocarbons with non-polar surfaces, which generally result in poor interfacial bonding with cementitious matrices. GF are composed mainly of SiO₂ and other oxides, and their surface is relatively rough, contributing to better mechanical anchorage [44,45]. the picture of the experimental material is shown in Fig 3.

The cost of the purchased materials is shown in Table 3.

**Table 3:** Fiber price list (Only for comparison of the cost of fiber improvement based on the current purchase price of the company).

PP	GF	PE	PET	SF
11 CNY/Kg	15 CNY/Kg	7.6 CNY/Kg	29 CNY/Kg	7.5 CNY/Kg

### Test method and test instrument

#### (1) Basic physical indexes of soil.

The primary physical indices of saline soil, including liquid limit, plastic limit, plasticity index, and specific gravity, were measured by JTG 3430–2020 [46]. A combined instrument for measuring both liquid and plastic limits was utilized.

#### (2) Compaction test of soil.

According to JTG 3430−2020 [46], the soil was crushed in the standard mould, and the demolding machine carried out the demolding. After demolding, the samples were wrapped and cured for 7 days.

#### (3) Experimental scheme.

**Fig 4:**
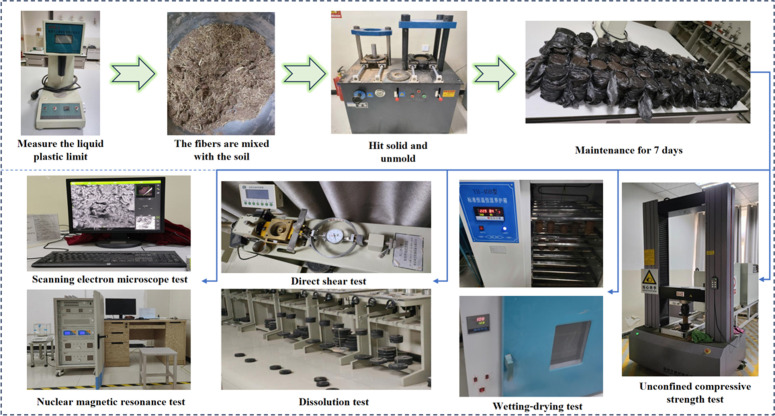
Experimental scheme diagram.

To investigate the effects of different synthetic fibers and their content on improved saline soil’s mechanical properties and micromorphology, a series of tests were conducted, including UCS, direct shear, wet-dry cycle, and dissolution tests. To further analyze the mechanisms underlying soil improvement, SEM and NMR experiments were performed. The fiber content used in the soil samples is listed in Table 4, and the experimental process is outlined in the accompanying Fig 4.

**Table 4:** Experimental scheme table.

Number	Type of fiber	Unconfined compression test	Direct shear test	Wetting-drying test	Dissolution test	SEM	NMR
1	0.5%PP + 4%SF	√	√				
2	1%PP + 4%SF	√	√			√	√
3	1.5%PP + 4%SF	√	√				
4	0.5%PP + 8%SF	√	√				
5	1%PP + 8%SF	√	√	√	√	√	√
6	1.5%PP + 8%SF	√	√				
7	0.5%PP	√	√				
8	1%PP	√	√				
9	1.5%PP	√	√			√	√
10	0.5%PET	√	√			√	
11	1%PET	√	√	√	√	√	√
12	1.5%PET	√	√			√	
13	1%PE	√	√				
14	2%PE	√	√		√	√	
15	4%GF	√	√				
16	5%GF	√	√				
17	6%GF	√	√				
18	7%GF	√	√	√	√	√	√
19	8%GF	√	√				
20	ST	√	√	√	√	√	√

Among the synthetic fibers tested, polypropylene fiber (PPF) is the most commonly used due to its high strength, heat resistance, acid and alkali resistance, and non-water-absorbent properties. Tiwari et al. [47] explored the addition of different percentages of steel fiber (SF) (2%, 4%, 8%) and PPF (0.25%, 0.5%, 1%) to strengthen expansive soil. Their results indicated that 0.5% PPF and 4% SF yielded the highest maximum dry density (MDD), while 1% PPF and 8% SF produced the highest shrinkage limit. Similarly, Alila et al. [48] studied the effects of PPF on expansive soil, revealing that increasing fiber content (0%, 0.2%, 0.4%, 0.6%, 0.8%) led to a 2.8% reduction in MDD due to lighter fibers replacing heavier soil particles. However, the optimal moisture content (OMC) remained largely unaffected as PPF does not absorb water. Based on these findings, PPF (0.5%, 1%, 1.5%) and SF (4%, 8%) were selected for this study.

Consoli et al. [49] conducted UCS tests using PETF contents of 0%, 0.50%, 1.00%, 1.50%, and 2.0%, revealing that UCS values increased with fiber content. Kumar et al. similarly found that injecting PETF into soft clay improved UCS, with optimal increases at 1% and 1.5% PETF; however, exceeding 2% led to a decline in UCS. Changizi and Haddad [50] demonstrated that polyester fiber was effective in stabilizing soil, with fiber contents of 0.1%, 0.3%, and 0.5% increasing CBR values by 1.2, 1.53, and 1.59 times, respectively, compared to unreinforced soil. Shear strength also improved with increasing fiber content, though the rate of improvement diminished at higher concentrations. Based on these findings, this study utilized 0.5%, 1%, and 1.5% PETF.

Hassan et al. [51] recently observed that including 1 cm PE cut from plastic bags enhanced CBR by 55% while adding PP improved CBR by 43%, with the highest UCS observed at 1% PE reinforcement. Choudhary et al. [52] investigated random soil reinforcement using varying amounts of high-density PE (0%, 0.25%, 0.50%, 1%, 2%, and 4%), finding that waste PE blocks significantly improved the deformation and strength of subgrade soil. The maximum enhancement in CBR and secant modulus occurred at 4%. Initially, this study designed experiments for 1% to 4% PE reinforcement using 2 cm plastic bags. However, due to the brittleness of the 3% and 4% samples, only 1% and 2% were used.

In another study, Kumar et al. [53] explored the stabilization of black cotton soil and loam (WCG) with recycled asphalt pavement (RAP) and broken glass, comparing results to red soil. They found that adding 65% RAP and 5% glass increased the CBR of black cotton soil from 2.71% to 9.68%, while 50% RAP and 5% glass enhanced the CBR of loam soil from 6.149% to 17.13%. Both soils outperformed red soil (3.96%). Mahdi and Al-Hassnawi [54] evaluated the impact of spiny glass powder in pavement subgrade, showing that 7% glass powder yielded the best CBR and UCS results. Therefore, GF fibers (4%, 5%, 6%, 7%, and 8%) were selected for this experiment.

#### (4) Unconfined compression test.

The specimens cured for seven days were subjected to unconfined compression using an electronic universal testing machine, allowing for compressive strength analysis across different fiber contents and observing failure modes. The machine had a maximum capacity of 10 kN, and the test was conducted at a 1 mm/min loading rate. The pressure and deformation of the soil samples were recorded throughout the experiment.

#### (5) Wet and dry cycle test.

After sorting and analyzing the experimental data of unconfined compression, the sample with the largest compressive strength among the five materials was selected for the dry-wet cycling test. They were treated in a constant temperature and humidity curing box at 20 °C and 99% humidity for 12h, then put into an oven at 108 °C for 6h. This was a dry and wet cycle process, and then the samples were compressed without lateral limiting after the dry and damp cycle. The morphology change and failure mode were observed.

#### (6) Direct shear test.

Direct shear tests were conducted on different soil samples under three loads of 50,150 and 250KPa. Shear was performed at a 1.2 mm/min shear rate, and displacements and dial indicator readings were recorded during shear.

#### (7) Dissolution test.

Five representative samples were tested using the consolidation device. Initially, the sample was placed on the consolidation apparatus, and a 1.0 KPa load was applied to ensure close contact between the sample and the parts of the consolidation instrument. The dial indicator was set to zero, and the preloading load was then removed. The dial indicator was read every 30 minutes at each load pressure stage until the deformation became stable (the stability criterion being that the deformation per hour does not exceed 0.01 mm). Once the deformation at the 200 KPa load level stabilized, fresh water was added to immerse the sample, and the deformation was monitored until it reached stability. Loading was continued step by step (increasing by 100 KPa per stage), with deformation being recorded at each level until stability was achieved. The dial indicator readings were recorded at regular intervals, and the dissolution coefficient of the saline soil was calculated using Formula (1). The dissolution test curve was then plotted.


$$\delta =\frac{h_{p}-h_{p}}{h_{0}}$$ (1)

(1) *δ*: Dissolution coefficient, calculated to 0.001;
(2) *h*_*p*_: Under P pressure, the height of the sample after stable deformation (mm);
(3) *h*_*p*_^*’*^: P pressure, the height of the sample immersed in water dissolution filter deformation after stability (mm);
(4) *h*_*0*_: Initial height of sample (mm);

#### (8) SEM test.

In order to study the microstructure of saline soil and analyze its curing mechanism, SEM was carried out. Before the experiment, the soil was made into a standard sample and glued to the slide. Subsequently, the SEM specimen was placed in an ion sputtering device for gold spraying to make the surface conductive. During the tests, the soil microstructure was observed using a Phenom ProX Generation 5 scanning electron microscope. Magnification and focal length were adjusted and images were taken when they were clear.

#### (9) Nuclear magnetic resonance test.

To judge the pore structure of saline soil more clearly, a nuclear magnetic resonance test was carried out on some samples, and the T_2_ distribution map of relaxation time was converted into the distribution map of pore structure by the Schlumberger-Doll Research equation.

## Analysis of experimental results

Maximum dry density (MDD), Optimal moisture content (OMC), Unconfined compressive strength (UCS), reinforced soil UCS/regular soil UCS (Kt), Cohesive force (C), Angle of internal friction (Φ), unconfined compressive strength after wet and dry cycling (UCS’), and dissolution coefficient (δ) of saline soil samples under different fiber contents are shown in Table 5.

**Table 5:** Experimental data table.

Number	Type of fiber	MDD (g/cm^3^)	OMC (%)	UCS (KPa)	Kt	C (10²KPa)	Φ (°)	UCS’ (KPa)	δ
1	0.5%PP + 4%SF	1.89	15.4	453.9	1.34	1.25	37.4		
2	1%PP + 4%SF	1.9	14	567.47	1.67	0.62	49		
3	1.5%PP + 4%SF	1.88	16.3	319.47	0.94	1.09	44.7		
4	0.5%PP + 8%SF	1.83	17	613.31	1.81	0.78	50.3		
5	1%PP + 8%SF	1.87	14.4	670.34	1.98	1.39	46.9	339.8	0.004
6	1.5%PP + 8%SF	1.81	16.6	518.67	1.53	1.49	42.2		
7	0.5%PP	1.98	12	526.68	1.55	0.91	49.1		
8	1%PP	1.96	13.8	467.43	1.38	1.32	36.3		
9	1.5%PP	1.94	12.5	553.71	1.63	0.92	46.3		
10	0.5%PET	2.24	11.4	418.83	1.24	0.74	49.4		
11	1%PET	1.94	13.8	485.4	1.43	0.78	49.7	1585.09	0.003
12	1.5%PET	1.96	14.2	378.2	1.12	0.31	51.3		
13	1%PE	1.93	14	285.32	0.84	−0.02	46.1		
14	2%PE	1.83	12.5	372.57	1.1	−0.07	49.7		0.005
15	4%GF	2.22	14	433.28	1.28	0.92	41.5		
16	5%GF	1.96	12	527.64	1.56	0.77	47.9		
17	6%GF	1.87	13.7	493.49	1.46	0.01	60.4		
18	7%GF	1.9	14.3	533.39	1.57	0.55	54.5	557.44	0.001
19	8%GF	1.88	14.4	339.59	1	0.63	48		
20	ST	1.94	13.8	338.97	1	1.88	26.6	989.75	0.005

### Compaction test results and analysis

**Fig 5:**
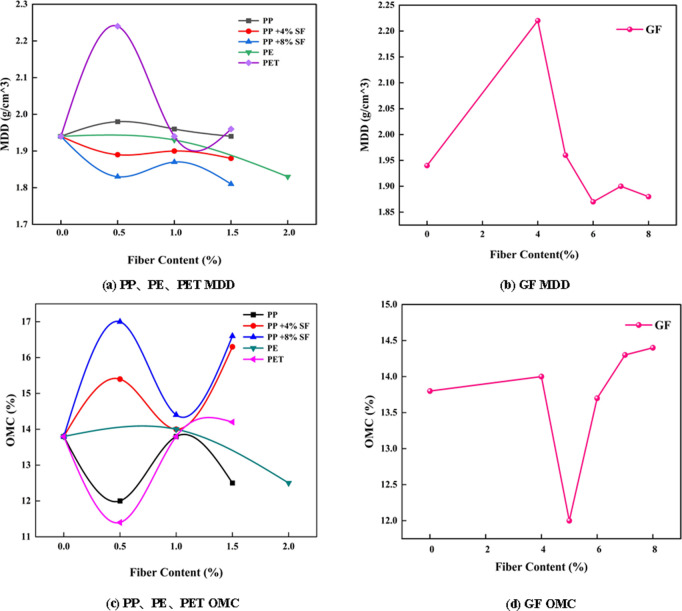
MDD and OMC of different fibers.

The MDD and OMC of different samples are shown in Fig 5.

Fig 5 shows that the MDD of soil reinforced with 4% and 8% SF, PPF, and PEF decreases to a certain extent compared to plain soil. Meanwhile, the OMC of soil reinforced with 4% and 8% SF and PPF increases, while PE fiber-reinforced soil decreases as fiber content increases. For GF-reinforced soil, the MDD initially rises and then falls with increasing fiber content, with the maximum MDD of 2.22 g/cm³ observed at 4% fiber content. As fiber content increases, OMC initially increases slightly, then decreases significantly before rising again. For PETF-reinforced soil, MDD first shows a marked increase and then decreases, while OMC initially drops significantly before increasing. The highest MDD of 2.24 g/cm³ and the lowest OMC of 11.4% are observed at 0.5% PETF content.

### Unconfined compressive strength test results and analysis

#### Stress-strain curve.

**Fig 6:**
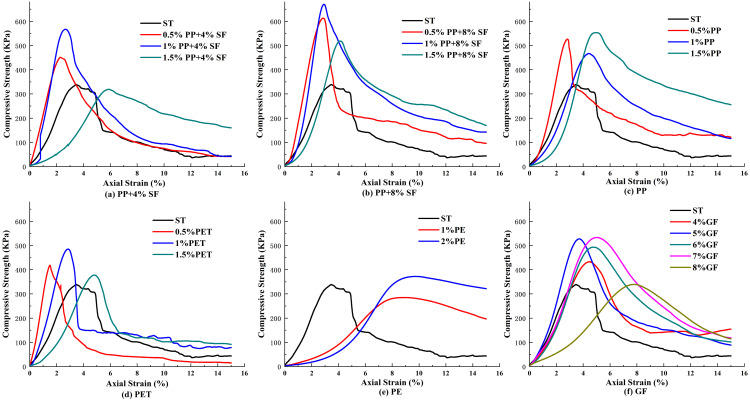
Stress-strain relationships of different fibers.

The electronic universal testing machine was used to carry out the unconfined compressive strength test of silty soil specimens, and the pressure and deformation of the specimens were recorded during the test. The stress-strain relationship obtained after processing the data is shown in Fig 6.

As shown in the Fig 6, the compressive strength of the reinforced soil with 1% polypropylene (PP) and 8% synthetic fiber (SF) is the highest, reaching 670.34 KPa, which is 1.98 times that of plain soil. When only polypropylene fiber (PPF) is added, the compressive strength initially increases, then decreases, and subsequently increases again with higher PPF content, aligning with the findings of Puppala, Musenda [55], and Alila et al. [48] The addition of PPF enhances the UCS of the soil. When SF and PP fibers are combined in saline soil, the UCS increases with rising SF content. At 4% and 8% SF content, the UCS increases and decreases with the PP fiber content, peaking at 1% PP. The trend observed with PETF is similar: as fiber content increases, the UCS initially rises before declining. The UCS of the reinforced soil with 1% PETF is the highest, at 1.43 times that of plain soil, corroborating the results of Changizi and Haddad [50].

Conversely, using polyethene (PE) plastic bags weakens the adhesive force between soil particles, creating larger voids that result in lower compressive strength than other fibers. The UCS of soils reinforced with 5% and 7% GF was 1.56 and 1.57 times that of plain soil, respectively, which is consistent with the findings of Mahdi and Al-Hassnawi [54]. The 7% waste glass powder achieved the best CBR and UCS values.

#### Failure mode of the sample surface.

**Fig 7:**
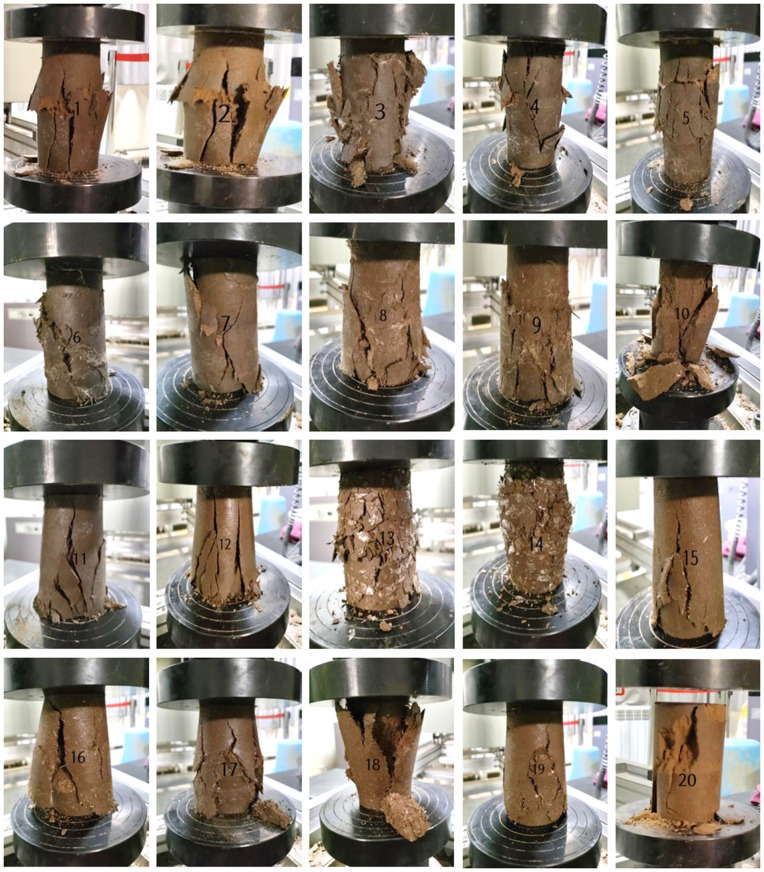
Failure patterns of different synthetic fiber-reinforced soil (The labels in the figure correspond to the serial numbers of the specimens in Tables 6 and 7).

The failure modes of saline soil modified by various synthetic fibers are illustrated in the Fig 7, with serial numbers corresponding to the test schemes. Notably, a vertical crack extends from the top to the bottom of the plain soil sample (specimen 20), displaying distinct shear bands. The dip angle of these shear bands ranges from 45° to 90°, and specimens 1 and 2 show some lateral expansion, with the soil mass warping along the shear bands. Numerous small cracks appear on the surface, attributed to the fibers enhancing adhesion between soil particles, effectively cementing the initially loose silty particles. This reinforcement of the soil particle skeleton improves overall integrity, delaying failure during the unconfined compressive strength test and providing better deformation resistance. Specimens 10–12, modified with varying PETF contents, exhibit long cracks from top to bottom, with shear band angles of 60° to 90°. Specimens 13 and 14, incorporating PEF, demonstrate significant ductility, featuring numerous small fine cracks and bulging in the horizontal direction. Lastly, specimens 15–19, reinforced with GF, show pronounced cracks under uniaxial compression, forming a shear failure surface with angles of 60° to 90°, while horizontal swelling is absent, indicating apparent axial failure.

### Wet and dry cycle test results and analysis

The wet-dry cycle test was carried out using ordinary soil and four kinds of fiber reinforced soil with the highest compressive strength as test materials, which were No.5, 11, 14, 18, and 20 specimens, respectively. PEF-reinforced soil was easily fractured during the experiment, so it was discarded, compacted, and cured for seven days according to JTG 3430−2020 [35] test procedures for highway geotechnical engineering.

#### Unconfined compressive strength.

**Fig 8:**
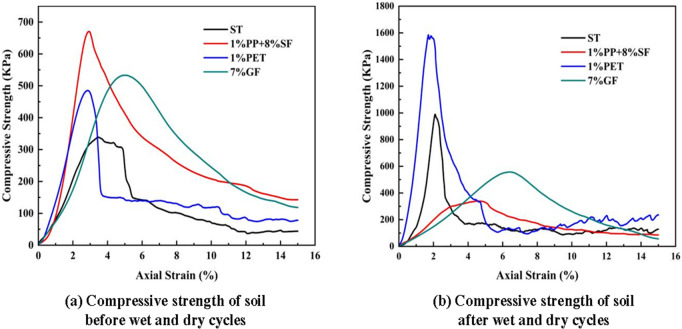
Changes in soil compressive strength before and after wet and dry cycles.

The compressive strength of the four specimens without dry and wet cycles is shown in Fig 8(a), and the unconfined compressive strength of the four specimens after three dry and wet cycles is shown in Fig 8(b).

Fig 8 indicates a significant improvement in the compressive strength of both soil and PETF-reinforced soil following wet-dry cycles. The UCS of PETF-reinforced soil reaches 1585.09 KPa, while the UCS of plain soil after these cycles is 989.75 KPa. The UCS values for GF and PPF-reinforced soils are 557.44 KPa and 339.8 KPa, respectively. The enhanced UCS of saline soil after wet-dry cycling can be attributed to the strengthening of the core structure of the solidified soil specimen. In contrast, the peripheral structure becomes weaker [56]. The saline soil contains a substantial amount of soluble salt, which migrates with water during the wet-dry cycle—dissolving when wet and crystallizing upon evaporation when dry. This crystallization creates a “cementing” effect between soil particles, improving compressive strength. The addition of PETF positively influences the performance of saline soil during wet-dry cycles by enhancing particle adhesion, limiting soil deformation, and improving overall integrity, which leads to increased strength of the reinforced soil. This suggests that PETF effectively mitigates soil deterioration during these cycles. In contrast, the UCS of GF is only marginally higher than that of soil not subjected to wet-dry cycles, potentially due to GF weakening the core reinforcement. Additionally, the reduced UCS of PPF-reinforced soil may result from fiber accumulation, weakening the bond between fibers and soil, and the fractures that occur during wet-dry cycling, which disrupt the cementing and bonding among soil particles.

#### Failure mode of the specimen.

**Fig 9:**
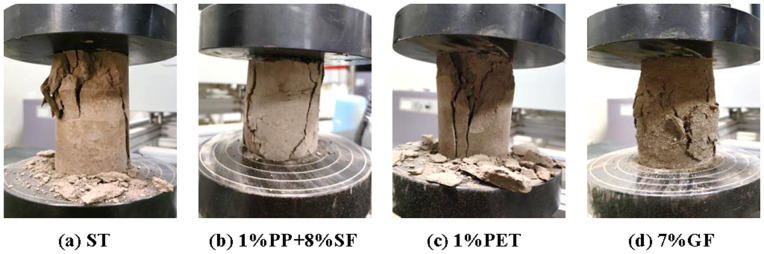
Breaking pattern of the specimen after wet and dry cycling.

It can be seen from Fig 9, after dry-wet cycling, the reinforced soil exhibits pronounced cracks under uniaxial compression, resulting in a shear failure surface with an inclination angle ranging from 60° to 90°. The GF-reinforced soil samples display horizontal swelling, while the axial failure is particularly evident in the other samples. However, the failure pattern of these reinforced samples is somewhat improved compared to the soil samples that did not undergo dry-wet cycling.

### Direct shear test results and analysis

**Fig 10:**
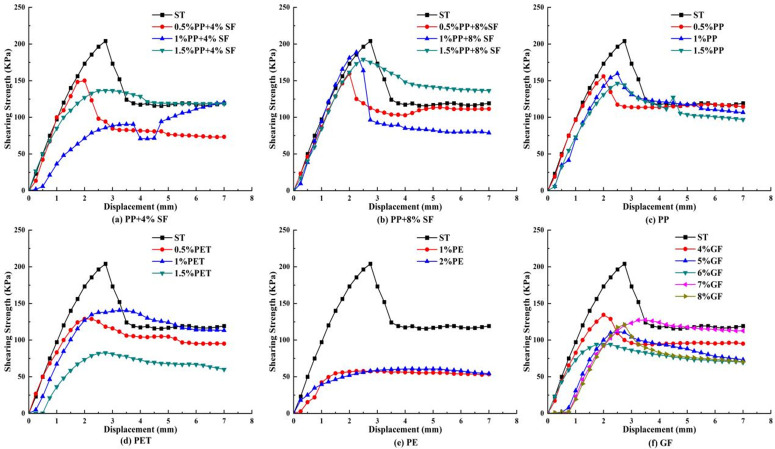
50KPa shear stress displacement relation curve.

**Fig 11:**
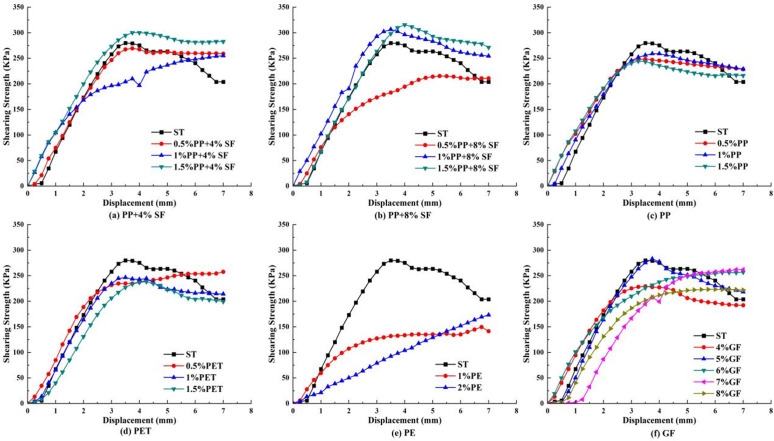
150KPa shear stress displacement relation curve.

**Fig 12:**
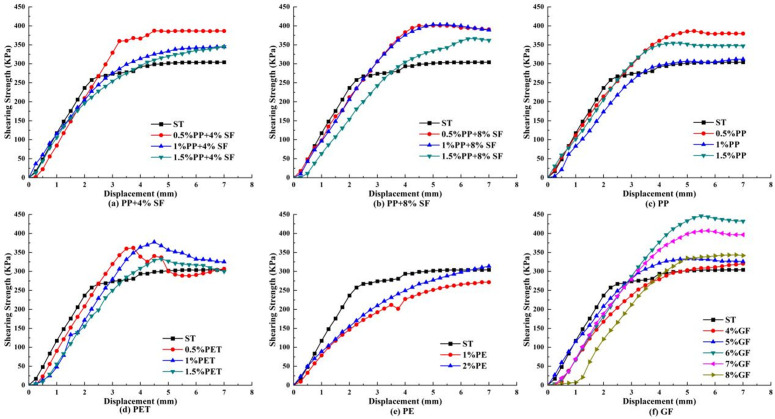
250KPa shear stress displacement relation curve.

The shear stress-displacement curve drawn from the displacement recorded by the direct shear test, which is the dial indicator reading, is shown in Figs 10–12.

As shown in Fig 10, the shear strength of plain soil exhibits a clear advantage under 50 KPa stress. With the addition of 4% SF, the shear strength of 0.5% PP exceeds that of both 1% and 1.5% PP. With the addition of 8% SF, the shear strength of 1% PP is superior to that of 0.5% and 1.5%. In the absence of silica fumes, the shear strength of 1% PP is higher than that of 0.5% and 1.5%, indicating that silica fumes significantly influences the shear strength of PP-reinforced soil. In PET-reinforced soil, the shear strength with 1% addition is slightly higher than with 0.5%, but much higher than with 1.5%, suggesting that excessive PET content may decrease shear strength. In contrast, the shear strength of PE-reinforced soil is low and consistently underperforms across different addition levels, while the shear strength of GF-reinforced soil is superior at the 4% addition level. These results indicate that different fiber types and addition ratios significantly affect the shear strength of reinforced soil, with silica fumes and fiber content playing crucial roles.

Fig 11 illustrates that the shear strength advantage of plain soil decreases significantly under 150 KPa stress. With the addition of 4% SF, the inclusion of 1.5% PP enhanced the shear strength of the reinforced soil, surpassing that of 0.5% and 1%. Similarly, with the addition of 8% SF, the inclusion of 1.5% PP exhibited better shear strength compared to the 1% and 0.5% additions. In the absence of silica fumes, the shear strength of 1% PP exceeds that of 0.5% and 1.5%, suggesting that silica fumes has a more pronounced enhancing effect on the shear strength of reinforced soil under high stress conditions. In PET-reinforced soil, the highest shear strength was achieved with the addition of 0.5%, and higher PET content did not further improve the shear strength. For GF-reinforced soil, when the GF content is 5%, its shear strength surpasses that of other addition levels. These results indicate that different fiber types and contents continue to significantly affect the shear strength of reinforced soil under high stress conditions, with the optimal addition ratios of silica fumes and fibers varying across different stress levels.

As shown in Fig 12, the shear strength of plain soil is significantly lower than that of reinforced soil under 250 KPa stress. With the addition of 4% SF, the inclusion of 0.5% PP improved the shear strength of reinforced soil compared to 1% and 1.5%. Similarly, with the addition of 8% SF, the inclusion of 0.5% PP also exhibited better shear strength than 1% and 1.5%. However, in the absence of silica fumes, the shear strength of 0.5% PP exceeded that of 1% and 1.5%, suggesting that a lower PP fiber content contributes more significantly to shear strength under high stress conditions. In PET-reinforced soil, the highest shear strength was achieved with the addition of 1%, slightly exceeding the 0.5% and 1.5% additions. However, the shear strength of PE-reinforced soil remains low, consistently under performing across all addition levels. For GF-reinforced soil, the shear strength is highest at the 6% addition level, surpassing that of other addition levels. This suggests that under 250 KPa stress, significant differences exist in the effects of various fiber types and their optimal addition levels on the shear strength of reinforced soil, with optimal additions of PP and GF fibers particularly enhancing shear strength.

Figs 10–12 show that as shear stress increases, the shear capacity of various materials gradually improves, and the peak displacement shifts to the right, indicating a significant enhancement in the deformation capacity of the soil under high shear stress conditions. Simultaneously, the residual shear stress in Fig 10 is the highest, while in Fig 12, it is the lowest, reflecting greater structural damage to the soil under high stress and a reduction in residual shear capacity. Additionally, with increasing shear stress, the shear capacity of all synthetic fiber-reinforced soils, except PE, improves significantly, indicating that synthetic fibers play a crucial role in enhancing shear resistance under high stress, demonstrating an excellent reinforcement effect.

**Fig 13:**
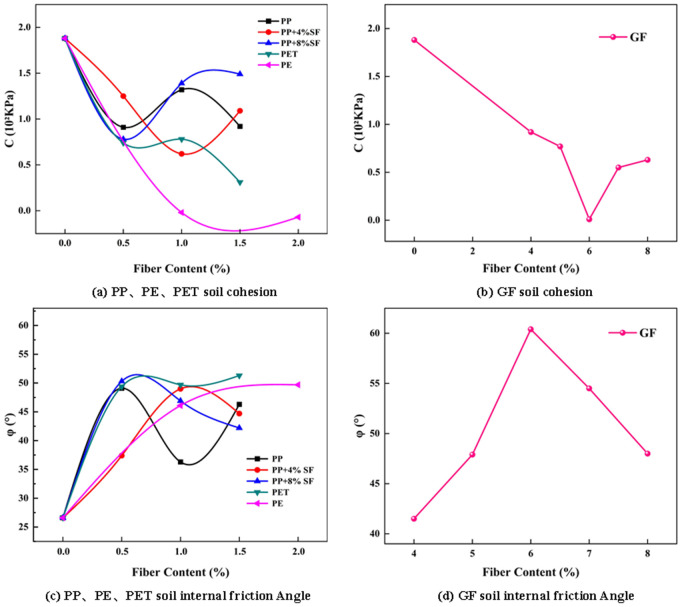
Cohesion and angle of internal friction of reinforced soils with different fiber contents.

The images of cohesion and internal friction Angle are shown in Fig 13.

Fig 13 shows that plain soil exhibits the highest cohesion without fiber addition, suggesting that fiber incorporation may disrupt the bonds between soil particles, particularly with PEF. However, the internal friction angle of the reinforced soil is significantly greater than that of plain soil, with shear strength in plain soil declining markedly as shear stress increases. The slope of the fitting curve for reinforced soil is steeper than that of plain soil. The internal friction angle is highest for 6% GF-reinforced soil, followed by 8% GF, 1.5% PET, 0.5% PP + 8% SF, 2% PE, and 1% PET, indicating that fiber addition enhances surface roughness and increases the soil’s resistance to shear failure, leading to more significant plastic deformation and a slower failure process. Regarding the influence of fiber content on cohesion and internal friction angle, when only PPF is added, cohesion initially decreases, then increases, and finally decreases again. The highest cohesion is observed in the reinforced soil with 1% PP, while the trend for the internal friction angle is inverse. For reinforced soil with PP and 4% SF, cohesion first decreases and then increases, peaking with 0.5% PP and again with PP and 8% SF. In PETF-reinforced soil, the highest cohesion is found at 1% PETF. Notably, PEF weakens the bonds between soil particles, damaging cohesion. In conclusion, under low stress conditions, cohesion is the dominant factor affecting shear properties, while under high stress conditions, the effect of internal friction Angle is more significant. Therefore, the 6%GF reinforced soil not only shows the optimal internal friction Angle under high stress conditions, but also has strong plastic deformation ability, which can effectively resist shear failure.

### Dissolution test of saline soil

**Fig 14:**
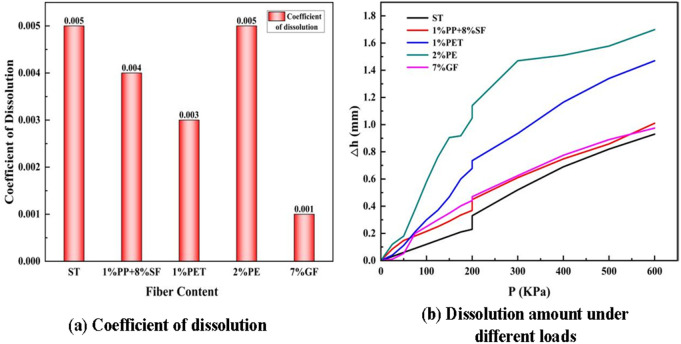
Dissolution test diagram.

Fig 14(a) illustrates that ST has the highest dissolution coefficient, reaching 0.005, which indicates its high susceptibility to collapse or subsidence when exposed to water. After the addition of synthetic fibers, the dissolution coefficient decreases, following this trend:

(1) 1%PP + 8%SF: The dissolution coefficient drops to 0.004, showing a slight improvement in soil stability compared to plain soil.1% PET: The coefficient decreases further to 0.003, suggesting that PET fiber provides better resistance to soil collapse than the combination of polypropylene and steel fibers.
(2) 2% PE: The dissolution coefficient remains unchanged from plain soil, indicating that polyethene fiber does not significantly enhance soil’s resistance to dissolution.
(3) 7% GF: The dissolution coefficient reaches the lowest value of 0.001, showing that glass fiber provides the most effective enhancement, significantly reducing the soil’s vulnerability to dissolution.

In Fig 14 (b), the settlement curve (Δh\Delta hΔh) shows that ST has a relatively low dissolution rate compared to some reinforced soils but continues to increase as the load rises. This suggests that, while plain soil initially resists the applied load, it becomes progressively more unstable as the pressure increases.

The curve for 1%PP + 8%SF closely follows that of plain soil, indicating only a moderate reduction in dissolution compared to the untreated soil. PET fiber significantly improves soil stability, with a lower slope in the curve, meaning the soil experiences less settlement under load. In contrast, the dissolution rate of 2% PE is much higher than that of the other fibers, indicating that polyethene fiber is less effective in reinforcing saline soil, allowing more deformation under higher loads. The settlement for GF-reinforced soil is the smallest, with a relatively flat curve, indicating that glass fiber provides the highest level of stability, minimizing the effects of load-induced dissolution.

Overall, the addition of fibers consistently reduces the dissolution coefficient, with glass fiber (7% GF) providing the most substantial improvement, followed by PET (1%) and PP + SF (1%PP + 8%SF). The same trend is observed in soil deformation under load, where GF significantly improves soil stability, followed by PET. On the other hand, polyethene fiber (2% PE) appears to be the least effective in resisting dissolution and reducing deformation under load.

### Scanning electron microscope test

**Fig 15:**
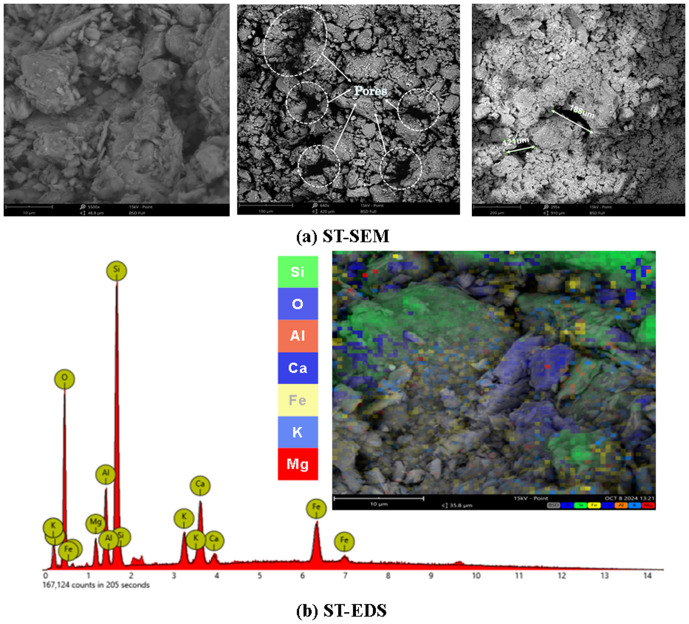
ST-SEM and ST-EDS.

In this experiment, a voltage of 15 kV was used for observation. The SEM image of the virgin soil, as shown in Fig 15(a), reveals a rough surface with numerous irregular pores, indicating a loose and weakly bonded microstructure. Fig 15(b) shows the ST-EDS image, Among them, Al, O, Si, K, Fe, Ca and other elements are abundant.

**Fig 16:**
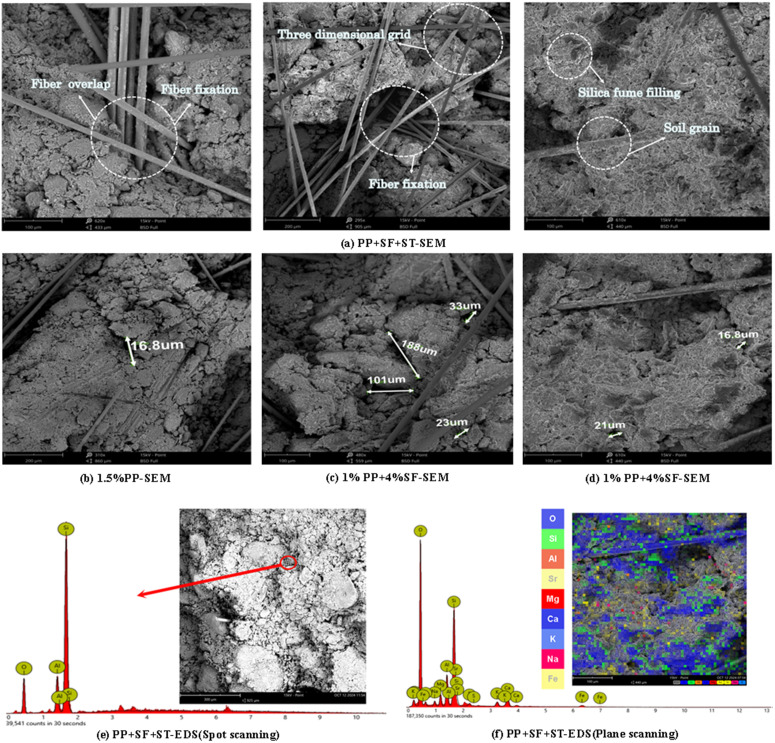
PP + SF-SEM and PP + SF-EDS.

Fig 16 (a) shows the SEM images of PP and SF-reinforced soil.

After fiber and silica fume addition, the SEM images show a clear embedding of PP fibers within the soil matrix, forming a cross-linked, grid-like reinforcement structure. This structure enhances mechanical interlocking and provides tensile bridging effects, which can resist crack propagation and improve load transfer under stress. Compared to the untreated soil, the samples with SF exhibit a more compact structure with smaller particles and a relatively smoother surface. Additionally, the incorporation of silica fume strengthens the bonding between the fibers and soil, as a soft layer adheres to the fiber surfaces, further enhancing the overall performance of the soil. Elemental analysis reveals a high silicon content in the soil-fiber interface, confirming the filling effect of the silica fume, Fig 16 shows the PP + ST-EDS image.As shown in Fig 16(e), the high Si content confirms that SF can form a strong bond with the soil. Fig 16(f) further reveals that the surface of PP fiber is enriched with elements such as O, Si, and Ca, indicating that the fiber surface is coated with a substantial amount of soil particles and SF. In conclusion, both PP fiber and SF effectively contribute to filling and protecting the soil matrix, consistent with prior studies [47,48].

**Fig 17:**
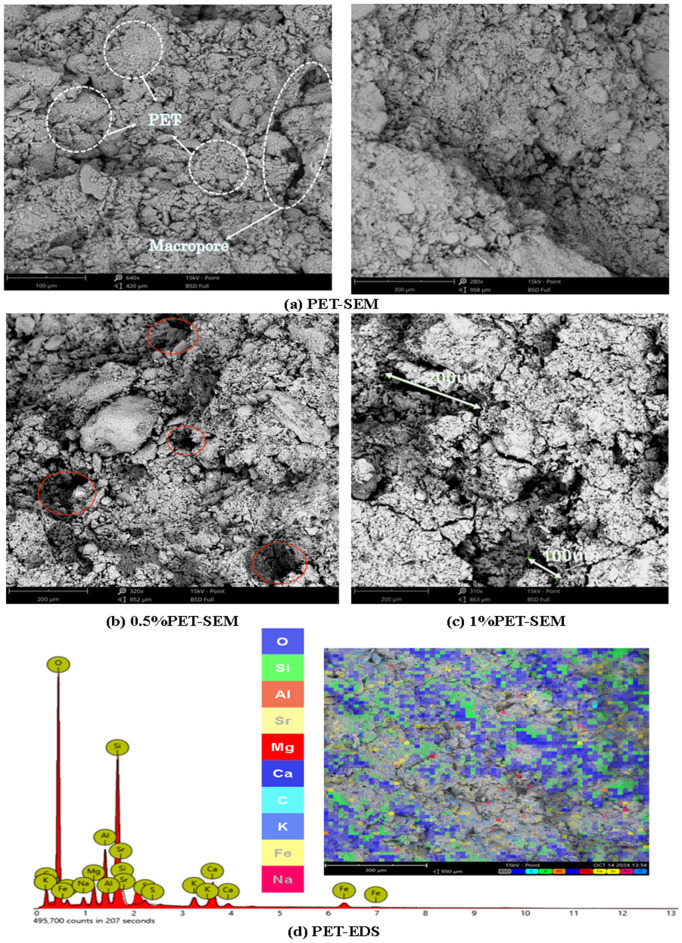
PET-SEM and PET-EDS.

Fig 17 shows the SEM image of PET-reinforced soil.

As shown in Fig 17(d), numerous small white particles are observed on the surface of the PET-reinforced soil. Due to the ultrafine particle size of PET fibers, they are unable to effectively fill the internal pores of the soil matrix. As a result, the composite exhibits a relatively loose structure characterized by a high number of large and interconnected voids, which could potentially compromise its mechanical integrity. Fig 17(d) shows the PET-EDS image, It can be seen that the content of O, Si, Al, Sr, K, C, Ca, and Fe is relatively abundant, it indicates that the distribution of fibers is relatively dispersed.

**Fig 18:**
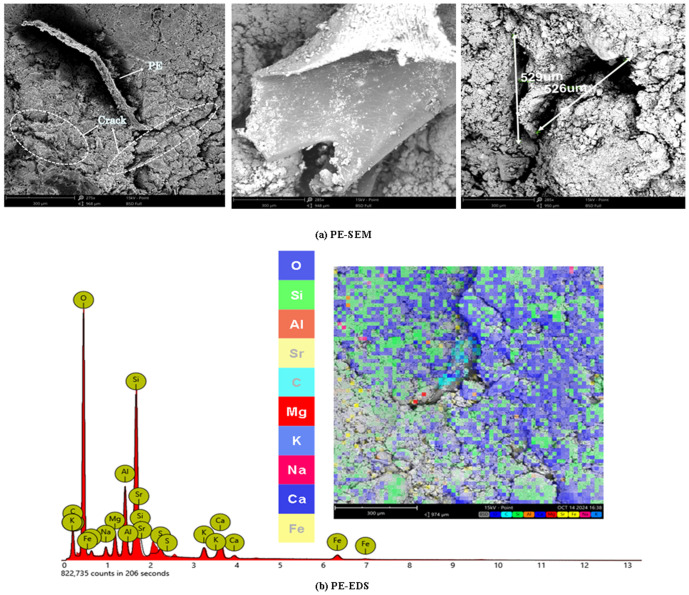
PE-SEM and PE-EDS.

Fig 18(a) shows the SEM image of PE-reinforced soil.

The poor bonding effect between PEF and soil may be because PEF itself is too large and can hinder the bonding between soil and soil particles, Fig 18(b) shows the PE-EDS image.

**Fig 19:**
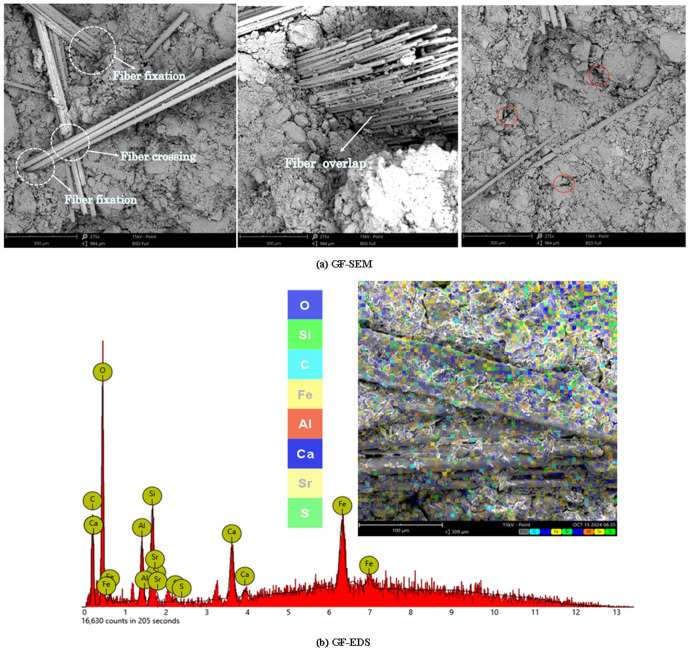
GF-SEM and GF-EDS.

Fig 19 (a) shows the SEM image of GF-reinforced soil.

The bonding effect between glass fiber and soil is good, and there is almost no gap between the fiber and soil. The staggered arrangement of glass fiber and the close gripping effect between glass fiber and soil can significantly enhance the mechanical properties of soil, Fig 19(b) shows the GF-EDS image, Fig 19(b) shows the high content of O, C, Ca, Si, Al and Fe in the reinforced soil.

### NMR microscopic analysis

#### Nuclear magnetic saturation analysis.

**Fig 20:**
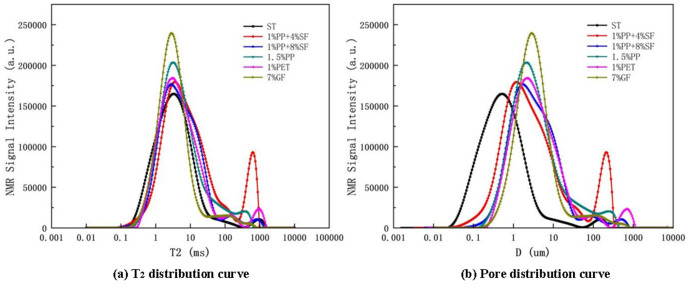
T2 distribution curve and Pore distribution curve.

The T_2_ time distributions of plain soil and reinforced soil are shown in Fig 20 (a).

At present, a large number of studies have shown that NMR technology can quickly and nondestructively evaluate the pore size distribution characteristics of porous media [57–62], the relaxation time (T_2_) can be obtained from equation (2) [63].


$$\frac{1}{T_{2}}=\;\frac{1}{T_{2B}}+\;\frac{1}{T_{2S}}+\;\frac{1}{T_{2D}}$$ (2)

Among them, T_2B_ is the free relaxation, T_2S_ is the surface relaxation, and T_2D_ is the diffusion relaxation. Since the pores of the soil are nano-scale, the surface relaxation plays a major role, and the free relaxation and diffusion relaxation can be ignored, so the following are:


$$\frac{1}{T_{2}}=\frac{1}{T_{2S}}=\rho \frac{S}{V}=\rho \frac{\alpha }{r}\;$$ (3)

Where ρ (um/us) is the surface relaxation coefficient, S is the pore surface area, V is the pore volume, and α is the geometric factor, depending on the shape of the pore. This study assumes that the pore structure consists of cylindrical pores, so α = 2.


$$\frac{1}{T_{2}}=\;\rho \frac{4}{D}$$ (4)

The Schlumberer-Doll Research (SDR) [64] equation is used to determine the surface relaxation rate coefficient, which requires T_2_ geometric mean T_2LM_, saturation permeability ks, and porosity Φ.


$$k_{s}=C\Phi^{4}T_{2LM}^{2}$$ (5)

$$k_{s}=\rho^{2}\Phi^{4}T_{2LM}^{2}$$ (6)

$$\rho =\sqrt{\frac{k_{s}}{\Phi^{4}T_{2LM}^{2}}}$$ (7)

After calculation, T2LM, ks and Φ of reinforced soil are shown in Table 6.

**Table 6:** NMR parameter table.

Number	T_2LM_(ms)	Φ	ρ(um/ms)
ST	3.524	0.268	0.0395
1%PP + 4%SF	1.191	0.291	0.0841
1%PP + 8%SF	0.814	0.294	0.1524
1.5%PP	0.934	0.283	0.1672
1%PET	0.598	0.268	0.1837
7%GF	1.512	0.283	0.2526

The Fig 20 (b) reveals that the soil treated with ST exhibits two distinct peaks, corresponding to small and large pores. The first peak represents pore sizes between 0.1 and 1 μm, while the second peak represents pore sizes between 100 and 1000 μm, showing a typical bimodal distribution (Fig 15(a)). After adding various fibers, the peaks of the pore size distribution shift to the right, indicating an overall increase in pore size in the reinforced soil. The distribution primarily concentrates within mesopores (1–10 μm) and macropores. Notably, the pore size distribution of the soil samples reinforced with PET fibers presents a three-peak structure, with pore sizes significantly larger than those in the other reinforced soils (Fig 17(b) and Fig 17(c)).

Regarding pore volume, reinforced soil shows considerable changes compared to plain soil. As the silica fumes content increases, the pore diameter in the reinforced soil initially decreases and then increases. In contrast, the pore volume follows an opposite trend—first increasing and then decreasing. This behaviour could be attributed to the fine particle size of silica fumes, which fills most of the soil pores, reducing the pore size(Fig 16(b), Fig 16(c) and Fig 16(d)). From Fig 17, PET-reinforced soil exhibits the largest pore size and volume, likely due to the small particle size of PET fibers, which are less effective in filling soil pores. This structure may compromise mechanical performance due to the presence of interconnected large pores, which serve as potential weak zones or water flow paths under external loading [65]. The GF-reinforced soil, on the other hand, contains a large number of mesopores but does not show a significant increase in total pore volume, suggesting that, while mesopores are abundant, the number of macropores and tiny pores is relatively lower(Fig 19(a)), This suggests that glass fibers promote the formation of uniformly distributed medium pores, contributing to better water retention and a more stable internal skeleton, which is beneficial for strength and durability enhancement.

#### NMR analysis before water saturation.

**Fig 21:**
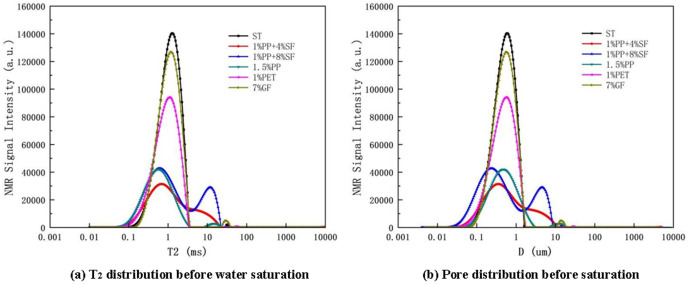
T2 distribution and pore distribution before water saturation.

The T_2_ distribution of plain soil and reinforced soil before water filling is shown in Fig 21(a).

After calculation, Before saturation, T2LM, ks and Φ of reinforced soil are shown in Table 7.

**Table 7:** NMR parameter table before water saturation.

Number	T_2LM_(ms)	Φ	ρ(um/ms)
ST	1.046	0.121	0.1156
1%PP + 4%SF	1.092	0.14	0.1282
1%PP + 8%SF	1.301	0.126	0.0969
1.5%PP	0.579	0.121	0.2089
1%PET	0.861	0.111	0.1289
7%GF	1.047	0.125	0.1193

The pore distribution curve is shown in Fig 21(b).

According to the NMR parameters and distribution map before and after saturation, T_2_ value increases significantly after saturation, which means that the increase of water has a large enhancement effect on the signal, while the pore size change may not be noticeable, so the effect of saturated water on the release of NMR signal is crucial.

## Conclusions

This study systematically examined the effects of four synthetic fibers—PPF, PETF, GF, and PE on the mechanical properties, collapsibility, and durability of saline soil. A comprehensive suite of mechanical, chemical, and microstructural tests was conducted to quantify the improvements. Based on the experimental results, the following key findings can be drawn:

(1) Compaction Behavior: The inclusion of PPF, PETF, and GF significantly increased the maximum dry density (MDD) of saline soil. Notably, 0.5% PETF and 4% GF resulted in the highest MDD values (2.24 g/cm³ and 2.22 g/cm³, respectively), approximately 1.6 times that of untreated soil. The lowest optimum moisture content (OMC) was observed in the 0.5% PETF-reinforced soil at 11.4%.
(2) Compressive Strength and Durability: Fiber reinforcement markedly improved compressive strength. The highest unconfined compressive strength (UCS) was achieved with 1% PPF + 8% SF (670.34 KPa), nearly doubling that of plain soil. The UCS of soils reinforced with 1% PETF, 5% GF, and 7% GF were 1.43, 1.56, and 1.57 times that of plain soil, respectively. After wet-dry cycles, UCS increased significantly for both untreated and PETF-reinforced soils, reaching 1585.09 KPa and 989.75 KPa, suggesting that such cycles are not necessarily indicative of saline soil failure. PPF exhibited the best overall compressive performance, followed by GF and PETF.
(3) Shear Strength Enhancement: Although fiber inclusion slightly reduced initial cohesion by disrupting inter-particle bonds, it enhanced shear resistance under increasing stress. Reinforced soils displayed steeper shear stress–strain curves than untreated soil. The highest internal friction angle was observed with 6% GF, followed by 8% GF, 1.5% PETF, 0.5% PPF + 8% SF, 2% PE, and 1% PETF. These results highlight the role of fibers in increasing surface roughness and promoting resistance to shear-induced deformation.
(4) Dissolution Resistance: All synthetic fibers effectively reduced the dissolution coefficient of saline soil, with GF showing the most pronounced improvement in both dissolution rate and total dissolution amount. This indicates enhanced durability and stability in saline environments.
(5) Micromechanical Mechanism: SEM and NMR analyses revealed strong interfacial bonding between fibers and soil particles, reduced porosity, and improved particle arrangement. These microstructural improvements explain the observed enhancements in macroscopic mechanical behavior. Elemental analyses further confirmed the presence and integration of reinforcement fibers in the soil matrix.

Among the tested fibers, glass fiber (GF) consistently delivered the best performance across multiple indices, making it the most promising candidate for reinforcing saline soils in practical engineering applications. The findings provide actionable guidance for fiber selection and dosage optimization in saline soil stabilization projects, particularly in arid and semi-arid regions where soil degradation poses a major engineering challenge.
